# Interdental papilla reconstruction: a systematic review

**DOI:** 10.1007/s00784-023-05409-0

**Published:** 2024-01-17

**Authors:** Monal Patel, Alaa Guni, Luigi Nibali, Ruben Garcia-Sanchez

**Affiliations:** 1 Periodontology Unit, Centre for Host Microbiome Interactions, King’s College London, Floor 18, Tower Wing, Great Maze Pond, London, SE1 9RT England, UK; 2 Periodontology Department Floor 25, Guy’s Tower Wing, Guy’s Hospital Great Maze Pond, London Bridge, London, SE1 9RT England, UK

**Keywords:** Interdental papilla reconstruction, Black triangles, Systematic review

## Abstract

**Objectives:**

To assess treatment options for the reconstruction of the lost interdental papilla and to evaluate evidence for their efficacy.

**Methods:**

An electronic search (Medline, Embase and the Cochrane Library Database and OpenGray) and a hand search were carried out to identify all types of studies investigating interdental papilla reconstruction (except for reviews) with a minimum of 3 months follow-up.

**Results:**

Forty-five studies were included in the study including 7 RCTs, 2 cohort studies, 19 case series and 17 case reports. Fifteen studies reported on the use of hyaluronic acid, 6 studies on platelet-rich fibrin, 16 studies on soft tissue grafting, 4 studies on orthodontics and 4 on additional modalities. The most common outcome measures were black triangle dimensions and papillary fill percentage. Meta-analysis was not possible due to the high heterogeneity of the studies.

**Conclusion:**

There are various options for interdental papilla reconstruction of which hyaluronic acid injections, PRF, surgical grafting and orthodontics seem to improve outcomes at a minimum 3 months. The use of soft tissue grafting with sub-epithelial connective tissue graft seems to be associated with the most robust evidence for the longer-term reduction of ‘black triangles’. There is insufficient evidence to make recommendations to clinicians. Further research is needed in the form of well conducted RCTs with longer follow ups and patient reported outcome measures.

**Clinical relevance:**

Patients frequently complain about the appearance of black triangles and their management options seem unclear. This systematic review provides insight into the available reconstructive options.

**Supplementary Information:**

The online version contains supplementary material available at 10.1007/s00784-023-05409-0.

## Introduction

The interdental papilla is an important anatomical part of the gingiva. It can reduce in height and can ultimately be lost due to a variety of causes which will be outlined in this introduction. This results in an open embrasure space commonly termed a ‘black triangle’. Black triangles can be highly unaesthetic and are a frequent cause of complaint by patients. An understanding of the available treatment options to manage this clinical issue is important for clinicians to ascertain.

### Anatomy of the interdental papilla

The interdental papilla is the part of the gingiva that fills the embrasure space between the contact points of adjacent teeth. It is supported by the underlying alveolar bone and laterally by the borders of the teeth [18]. It is comprised of masticatory mucosa and is composed of a dense connective tissue covered by oral epithelium [50]. The shape of the interdental papilla is influenced by the contact points between adjacent teeth, the width of the interproximal tooth surfaces and the course of the cemento-enamel junction (CEJ). The interdental papilla is pyramidal in shape at the anterior teeth. In posterior regions, there are two papillae joined by a concave saddle region called a ‘col’ [18]. The col can be either para-keratinised or non-keratinised [24].

The papillary height decreases from the anterior to the posterior teeth due to the interproximal contact area being most coronal between the central incisors and becoming progressively more apical along the arch. In contrast, the width of the col increases from the anterior to the posterior regions. The presence of the interdental papilla contributes to the scalloped shape of the gingival margin.

### Animal and human studies

In an experimental animal study investigating the anatomy of the interdental papilla, Kohl and Zander [30] investigated the effects of removing the interdental papilla in rhesus monkeys. In a split mouth design, they removed all interdental soft tissue to bone in two rhesus monkeys and after 2 months gently cleaned and polished the sites. The monkeys were sacrificed and specimens were prepared to study the interdental tissues. They found that the morphology of the interdental papilla confirmed Cohen’s description [17] and also concluded that the papilla and col reform to its original shape 8 weeks after the interdental tissues are removed. They also found that the col is non-keratinised and has a great deal of inflammation beneath it.

To assess this in humans, Holmes [24] conducted a human clinical study on 16 dental students. Specimens of excised interdental papillae were analysed. They found 30 out the 32 papillae had a concave shape in agreement with the findings of Cohen [18]. They also found that 22 out of the 32 papillae did not regenerate back to their original height after 32–86 days with gaps present in the embrasure spaces which is in contrast to the animal study by Kohl and Zander [30].

### Role of the interdental papilla

Historically, the function of the interdental papilla was thought to be only ‘deflection of food debris’. It was also theorised later that the interdental papilla could also have an important role as a barrier and defence to protect the underlying periodontal tissues [24]. A ‘round cell infiltration’ was found in the interdental papillae examined in specimens excised from a group of dental students. The inflammatory infiltrate demonstrates a defence mechanism to the constant threat of bacterial invasion from dental plaque accumulation.

**Fig. 1:**
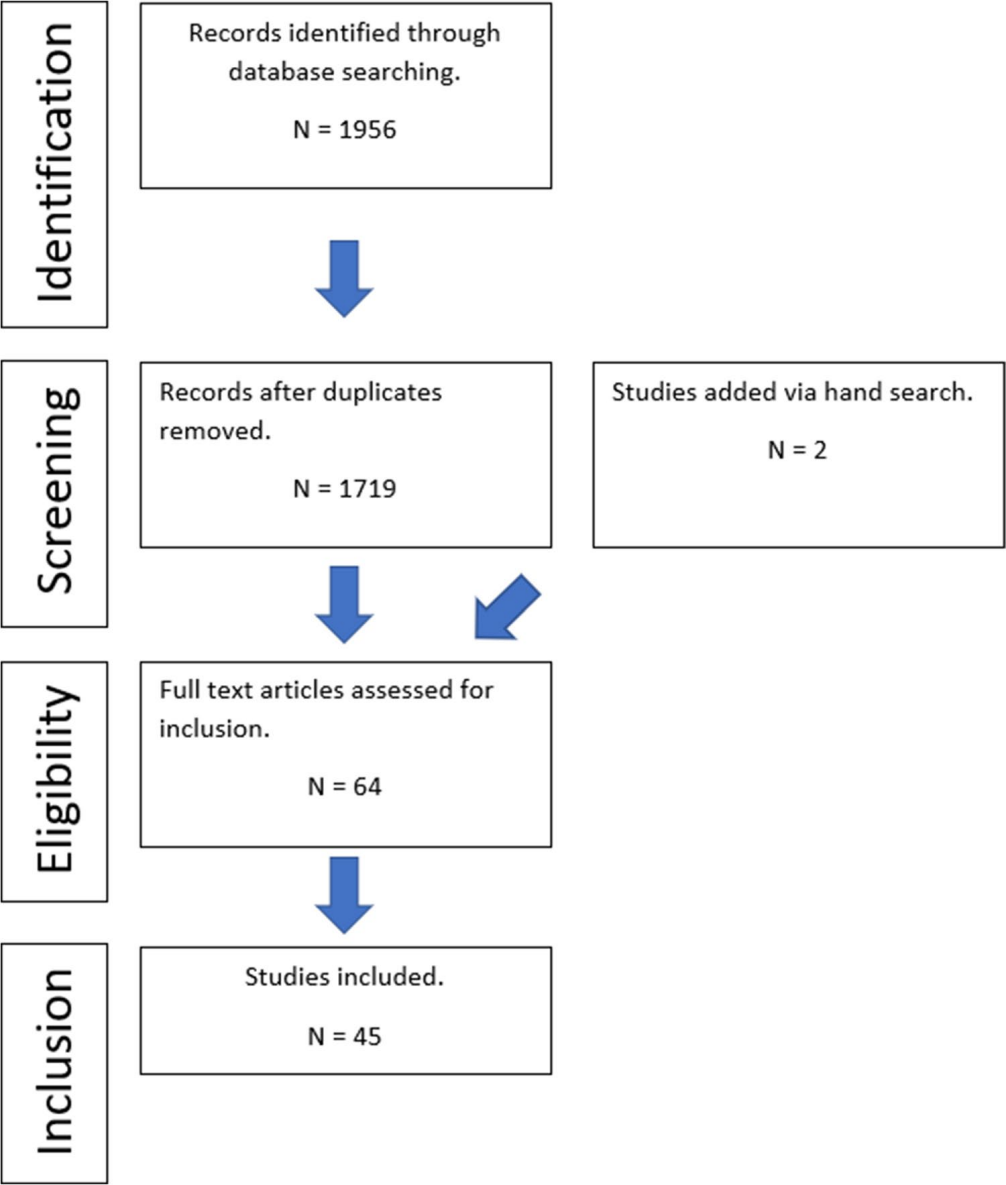
PRISMA flow diagram of the study selection process

The presence of the interdental papilla also plays an important role in aesthetics. A web-based study by Hochman et al. [23] investigated the layperson’s aesthetic preference of the interdental papilla in a low smile line. The participants were 200 lay people with no job connection to the dental field. They were shown three different professional medical illustrations of the lips and teeth with a low smile line. The first figure showed the presence of the interdental papillae (Fig. 1). The second figure showed an absence of interdental papillae and the presence of black triangles. The third figure showed an absence of interdental papilla with white restorations and long interproximal areas.

The participants were shown the illustrations via an online survey tool and asked to select the preferred illustration. The results of the survey found that 98% of the participants preferred the presence of the interdental papilla compared to the black triangles. Ninety-two percent of participants preferred the restored long contact area compared with the black triangles and 70% preferred the natural presence of interdental papillae compared to the absent interdental papillae with white restorations and long contact points.

This study demonstrates that even with a low smile line (which frequently is perceived by clinicians as being less challenging to treat), the absence of interdental papillae needs to be assessed in the smile analysis for restorative cases and that the clinical treatment of patients should include treatment options to manage missing interdental papilla.

This was a simple but effective study demonstrating how a lay person can perceive the presence or absence of the interdental papilla even in a low smile line. However, limitations were that illustrations were used rather than actual clinical photographs which are much more realistic. Also, 80% of the participants were Caucasian, and a more diverse population could potentially have led to different results.

### Factors affecting the presence of the interdental papilla

A clinical study by Tarnow et al. [66] investigated the relationship between the distance from the most coronal point of the interdental bone crest to the apical edge of the interdental contact point and the associated presence or absence of the interdental papilla. The authors used a large sample size of 288 sites in 30 randomly selected patients.

They found that as the distance (in millimetres) from the contact point to the bone crest increased, the presence of the papilla decreased. When the distance was 3–4 mm, an intact interdental papilla was present at 100% of sites. When the distance was 5 mm, an intact interdental papilla was present at 98% of sites. As the distance increased to 6 mm and above, there was partial or complete absence of the interdental papilla. For every millimetre increase, the chance of papilla presence reduced considerably. They concluded that the height of the interdental papilla is determined by the vertical height of the underlying bone.

In a clinical study, Chow et al. [16] studied 672 interproximal sites in 96 participants. Each interdental papilla was measured by a calibrated examiner and scored according to the [45] classification and also scored as either ‘competent’ or ‘deficient’. The participants’ age, gender, ethnicity and history of orthodontic treatment were recorded also.

They found that increasing age has an impact on the height of the papillae. They reported a 0.012-mm decrease in the height of the interdental papilla for every year in age. They also found that gingival thickness was related to the interdental papilla height. The presence of ‘competent’ papilla was associated with gingival thickness greater than 1.5 mm.

Joshi et al. [26] conducted a cross-sectional study assessing 150 interdental sites in 30 patients to assess factors associated with the extent of interdental papilla fill. They found that complete interdental papilla fill was significantly associated with tooth form or shape when the crown width-to-length ratio was greater than 0.88 and also when the bone crest to contact point distance of 5 mm or less. A higher gingival angle (measure of the gingival scallop) and an increased gingival thickness was significantly associated with competent papillae.

### Causes of loss of the interdental papilla

The interdental papilla can be lost due to interproximal bone loss due to periodontitis. The treatment for periodontitis can also lead to formation of black triangles. Both non-surgical therapy and surgical therapy to treat periodontitis, especially pocket elimination or resective surgery, will lead to reduction of loss of the interdental papilla. Episodes of necrotising periodontal disease can also lead to the formation of black triangles.

Iatrogenic damage such as over-contoured restorations and tissue damage from crown preparations can lead to the loss of interdental papilla. It can also be self-inflicted by the patient through traumatic brushing or overzealous use of interdental aids, pen chewing and piercings.

Tooth-related factors that can cause loss of the interdental papilla are as follows: loss of the contact point, tooth malposition, abnormal tooth shape, triangular-shaped crowns, diastemas, divergent roots and over-eruption of a tooth.

Orthodontic treatment can lead to loss of the interdental papilla. The prevalence of black triangle formation post-orthodontic treatment is reported to be 38% in adult patients [31].

A systematic review by Rashid et al. [52] aimed to assess the incidence of black triangles post-orthodontic therapy. Five studies were included and the incidence of black triangles following orthodontics was found to range from 38 to 58%. The authors reported that risk factors associated with the formation of black triangles were age, tooth-related factors, length of treatment and patient factors.

### Classification of the interdental papilla

There can be varying degrees of loss of interdental papilla height and so a classification system for this is a useful tool for clinicians and to allow standardised care. Classifications provide a basis for diagnosis, prognosis and subsequent management. They are useful for research purposes to allow homogeneity of data and allow integration of data for purposes such as meta-analysis for systematic reviews.

Nordland and Tarnow proposed a classification for the loss of interdental papillary height in 1998. It was based on three reference points: the contact point, buccal apical extent of the cemento-enamel junction (CEJ) and the interproximal CEJ. The classification is as follows:

- ‘Normal: the interdental papilla fills the embrasure space to the apical extent of the interdental contact point/area
- Class I: the tip of the interdental papilla lies between the interdental contact point and the most coronal extent of the CEJ
- Class II: the tip of the interdental papilla lies at/or apical to the interdental CEJ but coronal to the apical extent of the facial CEJ
- Class III: the tip of the interdental papilla lies level with or apical to the facial CEJ’

In 2004, Cardaropoli devised a newer classification of the interdental papilla height called the ‘Papilla presence index’ (PPI) with a scoring system from one to four. The classification is as follows:

- ‘Score 1: Papilla is completely present
- Score 2: Papilla is no longer completely present but the interdental CEJ is not visible
- Score 3: Papilla is no longer completely present and the interdental CEJ is visible
- Score 4: Papilla is no longer completely present. Both the buccal and interdental CEJ are visible’.

### Consequences of loss of interdental papilla

The loss of the interdental papilla can cause the appearance of black triangles which can be aesthetically displeasing and lead to food impaction and phonetic problems. This can lead to a negative impact in the oral health–related quality of life and self-esteem for the patient [49].

### Introduction to study

Due to its aesthetic impact, dentists often face a demand to try and manage or reconstruct the loss of interdental papilla. Treatment options for papilla reconstruction can be surgical, non-surgical or ortho-restorative in nature [22]. However, the management of papilla reconstruction is currently unpredictable and limited with several challenges faced and there is no consensus in terms of guidelines or treatment recommendations [54].

This systematic review on interdental papilla reconstruction aims to appraise the literature on the available treatment options to reconstruct the interdental papilla and evaluate how much evidence exists for the efficacy. The study aims to provide insight into the available treatment options and the strength of evidence for their use as a treatment option. It will allow clinicians to understand which options are available and to guide what further research is required to allow us to develop a protocol or guideline to manage loss of the interdental papilla.

### Aims and objectives

The aim of this systematic review was to systematically assess the treatment options available for the reconstruction of the lost interdental papilla and to evaluate evidence for their efficacy.

## Materials and methods

A protocol was developed in adherence to the PRISMA-P checklist (Preferred Reporting Items for Systematic review and Meta-analysis Protocols) based on [37] and the AMSTAR checklist (Assessing the Methodological Quality of Systematic Reviews) [60]. This systematic review was registered with PROSPERO (registration number CRD42021281184).

### Focused question

The main focused question of this review was as follows: ‘What are the available treatment options for reconstruction of the interdental papilla on natural teeth and how much evidence exists for their efficacy?’.

### Types of studies

For this systematic review, any type of human study ranging from case reports to randomised controlled trials were included.

### Eligibility criteria

The study selection criteria used in this systematic review were based on the PICOS method as follows:

- (P) The population was systemically healthy individuals with no age limit with loss of interdental papilla around natural teeth who underwent procedures aimed to reconstruct the interdental papilla.
- (I) Intervention: studies reporting on all forms of interventions aimed at reconstructing the interdental papilla were included.
- (C) Comparison: The control (if available) was a different type of intervention or no intervention.
- (O) Outcome variables: The following outcomes were evaluated:
  o Measurements of gingival level in the interdental papilla
  o Gingival recession
  o Probing pocket depth
  o Clinical attachment level
  o Bleeding on probing
  o Patient reported outcome measures relative to presence of the interdental papilla
- (S) Types of studies: any studies in humans (ranging from case reports, cohort studies to randomised controlled trials)

The inclusion criteria were as follows:

- Studies reporting treatment aimed at reconstructing the interdental papilla around natural teeth in humans
- Follow up of at least 3 months post-treatment

The following exclusion criteria were applied:

- Studies in animal models
- Reviews
- Studies focusing on dental implants
- Studies focusing on medically compromised patients
- Duplicate papers reporting data on the same sample and procedures as other publications

*Outcome variables*

The following outcomes were evaluated:

- Measurements of gingival level in the interdental papilla
- Recession
- Probing pocket depth
- Clinical attachment level
- Bleeding on probing
- Patient reported outcome measures relative to presence of the interdental papilla

### Risk of bias and methodological quality assessment

To assess the quality of the included studies, the risk of bias was independently evaluated by two reviewers (MP and AG). The assessment tool used to assess the risk of bias varied depending on the type of study design. For randomised control trials, the Cochrane Collaborations Tool was used in which seven domains were assessed for each study and categorised into high, unclear or low risk. For case–control and cohort studies, the Newcastle Ottawa Tool was used. For case series, the Modified Delphi tool was used and for case studies the CARE checklist was utilised to assess the quality of the studies. The levels of bias were categorised as low risk, unclear risk or high risk of bias based on the parameters of the various tools used. An assessment across all key domains were summarised and carried out by two reviewers (MP and AG) and any discrepancies were resolved by discussion.

### Search strategy

The search strategy involved searching the electronic databases Medline, Embase and the Cochrane Library Database. In addition to this, Open Grey search and manual search were also carried out with references of included papers and review articles also checked to determine any additional relevant papers. This included Journal of Clinical Periodontology, Journal of Periodontology, Journal of Dental Research and Journal of Periodontal Research. All papers up until October 2023 were included. There were no language restrictions applied on the initial search.

The electronic search strategy used the following key words and MESH terms:

dental papilla.mp. or exp Dental Papilla/

black triangle.mp.

interdental papilla.mp.

gingival recession.mp. or exp Gingival Recession/

treatment.mp. or exp Therapeutics/

management.mp.

reconstruction.mp.

regeneration.mp. or exp Regeneration/

repair.mp.

The study selection was conducted independently by two reviewers (MP and AG) and was completed in two phases.

Phase 1 involved the initial search involved screening relevant papers based on titles and abstracts that were potentially suitable and met the inclusion criteria. Any papers indicated as potentially suitable by at least one reviewer were included in the full text screening.

In phase 2, the full texts of potentially suitable papers were screened again. Any papers that did not meet the inclusion criteria were excluded at this point.

For any disagreements regarding the suitability of certain studies, reviewers tried to reach a consensus. In cases of continued disagreements, a third reviewer’s opinion (author RG) was sought for the final decision. After the full text screening, all suitable papers were added into a final database.

A data extraction spreadsheet was used to record data from the eligible studies. In particular, the following data was recorded:

- Study design
- Number of participants
- Population demographics, e.g. age, gender, ethnicity
- Smoking status
- Diagnosis of participants
- Control group (yes/no, what intervention if any)
- Type of intervention
- Variables measured
- Papilla indices
- Follow-up time
- Drop-outs
- Outcomes of the intervention
- Setting
- Funding
- Conflict of interest
- Ethics approval/informed consent

### Research synthesis and method analysis

Following the data extraction, the studies were analysed descriptively and similarities between the studies were determined and grouped together according to intervention type.

## Results

### Study selection

The initial search yielded a total of 1956 citations including 2 papers selected through a manual search. After analysis of the titles and abstracts and after removal of duplicates, 64 papers remained eligible for full text analysis. The full texts were screened and 45 papers met the inclusion criteria.

The kappa score was 0.89 for initial screening and 0.97 for final screening showing an excellent level of agreement.

A table that only includes papers that were excluded during phase two/full text screening was added to supplementary section (Table 1). The main reasons for exclusion were studies only presenting a description of a technique. One paper was a review.

**Table 1:** Study characteristics and outcomes for studies with hyaluronic acid

First author and year of publication	Study type Design Centre	Patient characteristics Number of patients Number of papilla defects Gender Mean age Smoking status		Intervention Control group if any	Treatment outcome assessment Follow-up
1) Abdeloraouf et al. [1]	RCT Parallel Cairo University Hospital—Single setting	10 patients 36 defects 7 female 3 male 32.55 years Non-smokers	2 No Yes	HA filler injection (Restylane lidocaine) repeated at 3 weeks and 6 weeks (4 patients 16 defects) Control—saline injection/placebo	Black triangle height PT-CP distance (distance from tip of papilla to contact point) (mean height ± standard deviation) Test − 0.25 ± 0.26 mm Control − 0.03 ± 0.13 mm Test group statistically significant reduction at 3 months but no significant change at 3–6 months Mean surface area of black triangle percentage change (± standard deviation) Test − 4.5 ± 28.5% Control − 2 ± 11.4% Patient satisfaction score (mean VAS score ± standard deviation) statistically significantly higher for test group 45 ± 12.65 vs control 27.65 ± 12.51 6 months
2) Ni et al. [44]	RCT Parallel Shanghai Ninth People’s Hospital, China Single setting	24 patients 68 defects 19 female, 2 male 41.3 years Non-smokers	3 Yes Yes	16 mg/ml HA gel injection, repeated at 3 weeks and 6 weeks Saline control group	Height of gingival papilla and area of black triangle, measured on clinical image Height of gingival papilla (mm ± standard deviation) Control 0.278 ± 0.45 Test 0.28 ± 0.38 Black triangle area (mm^2^ ± standard deviation) Control − 0.32 ± 0.50 Test − 0.450 ± 0.54 No statistically significant differences between groups, but HA group papilla increased in size earlier than the control group 12 months
3) Çankaya [68]	Cohort Single-arm open-label clinical trial Gazi university hospital, Turkey—single setting	20 patients 200 defects 10 female 10 male 34 years Non-smokers	0 No Yes	HA injections containing 2 mg/Ml HA non-cross-linked + 16 mg/mL HA gel (hyaDENT BG, BioScience) 3 injection points triangle, repeated at same points at 3 week intervals No control group	Interdental space area in mm^2^ ± standard deviation and percentage filling % ± standard deviation measured using digital impressions Mean interdental space area 0.06 ± 0.02mm^2^ Percentage filling 79.03 ± 4.98% 2 years
4) Alhabashneh et al. [3]	Case series/longitudinal study Prospective Jordan University of Science and Technology—single setting	27 patients 86 defects 17 female, 10 male Mean age not reported Non-smokers	6 No Yes	HA injection (HYADENT BG) following manufacturers 3 step protocol. Repeated at 3 weeks No control group	Black triangle height reduction (mm and %) using digital photos and probing measurements 3 weeks 0.17 mm 8% 3 months 0.83 mm 39% 6 months 0.62 mm 29% 6 months
5) Awartani and Tatakis [8]	Case series Prospective King Saud hospital, Saudi Arabia single setting	10 patients 17 defects 10 females 36.4 years 1 smoker (was excluded)	1 No Yes	HA gel injection repeated at 21 days and 42 days No control group	Percentage reduction of black triangle using clinical photographs and image analysis software Black triangle area 0.71 ± 0.74 Reduction 41 ± 37% 6 months
6) Kapoor and Dudeja [28]	Case series Prospective SGT university hospital, Delhi, India Single setting	6 patients 7 defects 4 female 2 male 37.5 years Non-smokers	0 No Yes	HA dermal filler gel injection, repeated at 3 weeks and 3 months No control group	Percentage gain in interdental papilla fill 88.2% Gain in interdental papilla height 3.3 mm 3 months
7) Lee et al. [32, 33]	Case series Chosun university hospital, South Korea Single setting	10 patients 43 defects 6 female 4 male 32 years Smoking status not reported	0 No Yes	HA gel injection (Teosyal Puresense Global ActionVR, Teoxane, Geneva) Switzerland). Repeated up to 5 times every 21 days until no black triangle visible	Interdental papilla reconstruction rate (%), decreases in black triangle area (mm^2^), width (mm), height (mm) Reduction in: Black triangle area 0.20mm^2^ Black triangle height 0.71 mm Black triangle width 0.32 mm Interdental papilla reconstruction rate 92.55% 6 months
8) Lee et al.[32, 33]	Case series Prospective Chosun University Hospital, South Korea Single setting	13 patients 65 sites [49] 7 female 6 male 32 years Smoking status not reported	8 sites excluded No Yes	HA gel injection repeated up to 5 times every 21 days until no black triangle visible	Interdental papilla reconstruction rate: percentage change of black triangle area Reduction in: Black triangle area 0.21mm^2^ Black triangle height 0.70 mm Black triangle width 0.30 mm Interdental papilla reconstruction rate 88.8% 6 months
9) Mansouri et al. [35]	Case series Islamic Azad University Hospital, Iran Single setting	11 patients 21 defects 8 females 3 males 37.5 ± 14.4 years Non smokers	6 months No Yes	HA injections repeated 3 times every 3 weeks	Gingival embrasure space calculated using clinical photographs based on pixel size using a formula Increase in papilla reconstruction ranging from 20–100% with mean (percentage ± standard deviation) 47.33 ± 20.20% at 6 months
10) Ni et al. [43]	Case series Prospective Shanghai Ninth People’s Hospital, China Single setting	8 patients 22 defects 8 females 41.6 years Non-smokers	0 Yes Yes	HA gel injection, repeated at 3 weeks and 6 weeks No control group	Clinical and photographic measurements and DIGIMIZER, height of gingival papilla and area of black triangle Height of papillae increased by 0.4 mm Area of black triangle reduced by 0.36mm^2^ 12 months
11) Patil [47]	Case series Maharashtra University Hospital, India Single setting	8 patients 5 female 3 male 32 years Smoking status not reported	0 No Yes	HA dermal filler injection, repeated at 3 weeks if dark space remained repeated up to 3 times	Black triangle height and width, pixel values clinical/photographs. Partial or complete papilla reconstruction 8 sites complete papilla reconstruction achieved 6 sites partial reconstruction with IPRR ranging from 16–91% Mean reduction in: Black triangle area: 0.25mm^2^ Black triangle height: 0.85 mm Black triangle width: 0.34 mm 3 months
12) Pitale et al. [49]	Case series Modern Dental College and Research Centre, Madya Pradesh, India University hospital—single setting	7 patients 25 defects 5 females, 2 males 30.96 years Non-smokers	Not mentioned No Yes	0.2 ml HA injected and massaged for 2–3 min GENOSS®	Papillary recession height CP-GM and interproximal width, photo analysis—black triangle height and width CP-GM 0.64 mm Black triangle height 0.64 mm Interproximal width 0.44 mm Black triangle width 0.43 mm 6 months
13) Singh and Vandana [63]	Case series Prospective University hospital Karnataka, India Single setting	10 patients 42 defects Gender numbers not specified Age range 25–40 years Non-smokers	1 patient (7 defects) No Yes	1% HA of group (16 sites), 2% of HA group (7 sites) and 5% of HA group (12 sites) repeated at 2nd and 3rd weeks	Clinical and photographic measurements-reduction in black triangle space 6 months 5% HA showed 18.2% clinical enhancement and 39.8% photographic improvement at 6 months Intergroup comparison non-significant
14) Spano et al. [64]	Case report University of Toronto, Canada Single setting	3 patients 4 defects 3 females 51.7 years Non-smokers	0 Filler donated by Allergan, Canada Yes	Subperiosteal papilla augmentation with non-animal derived hyaluronic acid overlay technique	Mean papilla fill (mm ± standard deviation)1.75 mm ± 0.5 VAS score: 62.5% improvement in perception of papilla 6 months
15) Tanwar and Hungund [65]	Case report Darshan Dental College and Hospital, Udaipur, India—single setting	1 patient 1 defect 1 female 24 years Non-smoker	0 No Yes	< 0.2 ml HA gel injection repeated at 3 weeks	Measurements of black triangle using photographs Significant gain in papillary volume at 3 months

### Study design and population

Of the 44 papers included in the study, there were 7 randomised controlled trials [1, 2, 12, 37, 38], 2 cohort studies [3], 19 case series [4–10, 13, 20–22, 24, 25] and 17 case reports [11, 14–16, 27–29, 31–33, 35, 39, 40].

Fifteen papers reported on the use of hyaluronic acid [1–11], 6 papers reported on the use of platelet-rich fibrin (PRF) [12–15], 16 papers on the use of various grafting techniques [16, 20–22, 24, 25, 27–29, 31–33], 4 studies on orthodontics [34–36] and 4 studies on different modalities [37–40]. The study setting varied from various locations around the world from Asia, the Middle East, South America and Europe. Thirty-seven studies took place in university hospital settings whilst 2 studies took place in private practice settings and for 3 studies the setting was not clear. All studies were in single settings and there were no multicentre studies.

The study population ranged from 1 to 143 patients and the number of defects reported ranged from 1 to 200. The follow-up times reported ranged from 3 months to 7 years. In one study [57], a smoker was included in one of the case reports whilst 28 studies excluded smokers and 14 studies did not report of smoking status of the participants.

The outcome measures varied between studies but most commonly included black triangle height, width and surface area, percentage fill or reduction in black triangle area and change in papillary fill. Two studies by Lee et al. [32, 33] also reported the interdental papilla reconstruction rate. Three studies [22, 32] reported outcomes with change in PPI scores. One study [62] reported on the ‘papilla esthetic score’ (PES) as an outcome. Four studies [25, 28, 29] did not give numerical outcomes but reported the visual appearance of the papillae. Visual analog scores were reported for 4 studies [1, 12, 38].

Tables 1, 2, 3, 4 and 5 report a description of studies with study outcomes divided according to modality of papilla reconstruction as follows:

1. Hyaluronic acid (HA) (Table 1)

**Table 2:** Study characteristics and outcomes for studies with PRF

First author and year of publication	Study type Design Centre	Patient characteristics Number of patients Number of papilla defects Gender Mean age Smoking status	Dropouts Funding Ethical approval	Intervention Control group if any	Treatment outcome assessment Follow-up
16) Sharma et al. [59]	RCT Parallel University hospital Uttar Pradesh, India Single setting	20 patients 20 defects 18–50 years Non-smokers	0 No Yes	Han and Takei technique using SCTG Han and Takei technique using PRF	Reduction in CPTP distance and mean gain in papillary height SCTG group better papillary fill achieved compared to PRF PRF group less morbidity 3 months
17) Singh et al. [62]	RCT Parallel Saraswati Dental College, Lucknow, India Single setting	40 patients 40 defects Similar gender match 30.57 years Non-smokers	0 No Yes	Surgery with PRF Control group connective tissue graft	Papilla height (mm), VAS score (%), PES score 3 months Increase interdental papilla height 3.10 mm group 1, 3.45 mm group 2. Complete papillary fill group 1: 90% group 2: 95% Patient satisfaction higher group 1
18) Ahila et al. [2]	Case series Prospective Indira Gandhi Institute of Dental Sciences, Puducherry, India Single setting	13 patients 25 defects Male and females in sample but exact details not specified Mean age not given age range 18–55 years Non-smokers	0 No Yes	Papilla augmentation (Han and Takei technique) with PRF	Distance from tip of papilla to contact point: reduced from 4.38 mm to 0.36 mm, mean papilla fill, increase in width of keratinised gingiva, Jemt score, and visual analog scale statistically significant 6 months
19) Arunachulam (2012)	Case report Thai Moogambigai Dental College and Hospital, India	1 patient 2 defects 1 female 40 years Non-smoker	0 No Not mentioned	Pedicle flap and PRF	Papilla contour measurements based on PIS score PIS conversion from 1 to 3 6 months
20) Vijaylakshmi et al. [69]	Case report University hospital, Chennai, India Single setting	1 patient 1 defect 1 male 37 years Smoking status not reported	0 No Yes	Modified beagle's technique with A-PRF membrane	Papillary fill percentage 100% PPI went from 2 to 1 3 months
21) Yamada et al. [70]	Case reports University hospital Japan Single setting	10 patients 10 defects 7 female 3 male 39 years Smoking status not reported (5 cases on implants, 5 cases on natural teeth)	0 Not mentioned Yes	Tissue engineered papilla injections: combination of bone marrow–derived mesenchymal cells with platelet-rich fibrin and hyaluronic acid mixed with thrombin and calcium chloride Injected adjacent to papilla	Decrease in black triangle height—distance from tip of papilla to the base of the contact area Mean value (mm ± standard deviation) of improved black triangle 2.55 ± 0.89 mm 27–69 months

**Table 3:** Study characteristics and outcomes for studies with soft tissue grafting

First author and year of publication	Study type Design Centre	Patient characteristics Number of patients Number of papilla defects Gender Mean age Smoking status	Dropouts Funding Ethical approval	Intervention Control group if any	Treatment outcome assessment Follow-up
22) Chacon et al. [14]	Case report Private practice Colombia	1 patient 1 defect 1 female 24 years Non-smoker	0 No No	Subepithelial connective tissue graft No control group	Visual assessment 18 months
23) Chaulker et al. [15]	RCT Parallel University hospital Pune, India Single setting	Number of patients not clear 20 defects Age range 20–50 years Non-smokers	0 No Yes	Test: Modified Beagle’s technique Control: Beagle’s technique	Vertical papillary defect height, mesiodistal papillary defect width, total papillary defect area. All reduced in group A and slightly increased in group B 6 months
24) Feuillet et al. [20]	Case series Not clear- private practice?	3 patients 3 defects 1 female, 2 males Range 37–45 years Non-smokers	0 Not mentioned Yes	Interproximal tunnelling with customised connective tissue graft No control group	Embrasure fill PPI score Case 1: From 2 to 3 Case 2: From 1 to 3 Case 3: From 1 to 2 2 years
25) Kaushik et al. [29]	Case series Prospective university hospital Ambala, India Single setting	10 patients 15 defects Gender not mentioned Inclusion age range 8–55 years Non-smokers	0 No Yes	Subepithelial connective tissue graft No control group	Distance from contact point to gingival margin mean difference 0.80 mm ± 0.94 (not stat significant) 6 months
26) Nemcovsky (2001) [42]	Case series Tel Aviv university hospital, Israel Single setting	9 patients 10 defects Gender not reported 39.9 years Non-smokers	0 No Yes	Surgical papilla augmentation using advanced papillary flap combined with gingival graft	Change in papilla contour measurements and PIS score PIS increased in 8 procedures The difference between pre- and postoperative PIS ranged between 0 and 3 PIS units (mean PIS unit ± standard deviation) 1.2 ± 0.92 At 3 months
27) Muthukumar (2011)	Case series Sri Ramachandra university, India Single setting	3 patients 3 defects 1 female 2 males 24-year-old female (ages of others unreported) Smoking status not reported	0 No Yes	Case 1: Han and Takei technique and orthodontics post op Cases 2 and 3: modification of the Nordland technique	Complete reconstruction of papilla assessed by clinical/visual assessment at 1 year
28) Sawai and Kohad [56]	Case series Prospective Government dental college, India Single setting	19 patients 39 defects 12 female 7 male 29 years Non-smokers	0 No Not mentioned	Variant of Beagle’s technique	Improvement in contour in 51% of cases and in 38.46% the interdental papilla completely obliterated the open embrasure space 6 months
29) Sharma et al. [58]	Case series Prospective Oxford dental college hospital Bangalore—India—single setting	11 patients 6 females 5 males Over 18 years Non-smokers	0 Not mentioned Yes	Subepithelial connective tissue graft No control group	Vertical and horizontal component and area of black triangle 23.3% reduction in horizontal component from baseline to 3 months and 37.7% from 3 to 6 months Mean area of black triangle decreased to 0.92mm^2^ at 6 months
30) Shenoy et al. [61]	Case series Univeristy hospital, Karnataka, India Single setting	3 patients 3 defects 2 female 1 male Age range 30–45 years Smoking status not reported	1 Not mentioned Not mentioned	Cases 1 and 2: roll technique Case 3: connective tissue graft and tunnel technique	Papilla presence score decreased in all three cases Visual assessment 6 months–2 years
31) Azzi et al. [9]	Case report Unclear—private practice?	1 1 Female 57 years Non-smoker	0 Not mentioned Not mentioned	Coronally advanced flap with sub-epithelial connective tissue graft No control group	At 3 months: Complete root coverage on facial aspect of central incisor and canine, 1 mm residual recession UR2, soft tissue margin 4 mm more than at baseline 18 months
32) Beagle [10]	Case report Setting unclear	1 1 1 female 28 years Smoking status not reported	0 Not mentioned Not mentioned	Surgical reconstruction of the interdental papilla using a combination of Abram’s roll technique for ridge augmentation and Evian’s papilla preservation technique No control group	Visual clinical outcome Improved cosmetics, stayed stable at 18 months with slight recession
33) Carnio [12]	Case report Private practice—single setting	1 1 1 female 20 years Non-smoker	0 No Not mentioned	Interposed subepithelial connective tissue graft	Interpapillary space was completely filled, height and volume maintained, previous buccal recession covered at 4 years
34) Jaiswal et al. [25]	Case reports Sharad Pawar Dental College, India-University hospital Single setting	5 patients 5 defects 3 females, 2 males 36.2 ± 10.37 years Non-smokers	0 No Yes	Subepithelial connective tissue graft and coronally advanced flap No control group	Papilla contour measurements (mm ± standard deviation) Vertical distance decreased from 3.2 ± 0.44 mm (initial) to 0.4 ± 0.54 mm at 6 months
35) Muthukumar et al. [39]	Case report Sri Ramachandra hospital, India University hospital—single setting	1 patient 1 defect 1 male 26 years Smoking status non reported	0 No Not mentioned	Autogenous bone and connective tissue graft No control group	Surface area, perimeter and length of lost interdental tissue using image analysis software Lost interpapillary space length 5–2.7 mm, surface area 88 to 20mm^2^, perimeter 12.1 to 5.5 mm from pre to post op 6 months
36) Palathingal and Mahendra [46]	Case report Meenakshi Ammal Dental College and hospital, India University hospital—single setting	1 patient 1 defect 2 males 21 years Smoking status not reported	0 Not mentioned Yes	Semi lunar coronally repositioned papilla technique with subepithelial connective tissue graft	Papilla index score, height of interdental papilla PIS increased from 2 to 3 height increased from 3 to 4 mm at 180 days
37) Pinto et al. [48]	Case reports University of Sao Paulo, Brazil Single setting	2 patients 2 defects 2 females 33.5 years Non-smokers	0 No Yes	Subepithelial connective tissue graft with coronally advanced flaps	Root coverage percentage: 3.5 mm coverage (87.5%) for lateral incisor 3 mm coverage (100%) for canine Distance from tip of papilla to contact point decreased by 3.5 mm (case 1) and 1 mm (80% reduction) for case 2 4 months to 1 year

**Table 4:** Study characteristics and outcomes for studies with orthodontics

First author and year of publication	Study type Design Centre	Patient characteristics Number of patients Number of papilla defects Gender Mean age Smoking status	Dropouts Funding Ethical approval	Intervention	Treatment outcome assessment Follow-up
38) Kandasamy et al. [27]	Cohort study Private orthodontic practice—Western Australia Single setting	143 patients Defect number unclear 83 females 60 males Age range 13–16 Smoking status not reported (assumed non-smokers)	0 Funded by university research fund Yes	Orthodontic treatment Control: sample of 25 patients with well aligned teeth	Percentage increase of decrease in interdental papilla heights pre and post treatment 18 months Height of interdental papilla decrease when teeth move labially and height of papilla between teeth increased when teeth were intruded to a palatal position
39) Cardaropoli and Re (2005)	Case series Prospective Not clear—private practice?	28 patients 28 defects 22 female, 6 male 44.79 years Smoking status not reported	0 No Not clear	Open flap debridement and orthodontic intrusion	PPD, CAL, PI score Mean PPD 2.50 mm mean CAL 5.93 mm, 23/28 patients had improved PI score 1 year
40) Carnio and Carnio [13]	Case report Prospective Private practice—Brazil—single setting	1 patient 1 defect 1 female 48 years old Smoking status not reported	0 No Not clear	2 surgical soft tissue grafts and orthodontics	Papilla height, appearance of papillary architecture 3-mm gain of papilla height, correction of reverse papilla architecture, interproximal soft tissue at level of CEJ 10 years
41) Sato et al. [55]	Case report Setting unclear University hospital, Tokyo, Japan Single setting	1 patient 1 defect 1 female 58 years Non-smoker	0 Not mentioned Not mentioned	Periodontal treatment followed by orthodontic treatment using light force and simultaneous mesial stripping of the incisors	Visual clinical assessment Formation of non-surgical papilla 1 year

**Table 5:** Study characteristics and outcomes for studies with additional modalities

First author and year of publication	Study type Design Centre	Patient characteristics Number of patients Number of papilla defects Gender Mean age Smoking status	Dropouts Funding Ethical approval	Intervention	Treatment outcome assessment and results Follow-up
42) Çankaya et al. [11]	RCT Prospective Gazi University, Turkey Single setting	40 patients 120 defects 20 female 20 male 25–45 years Non-smokers	13 0 Yes	Surgical papilla regeneration with concentrated growth factor (CGF) Control—no surgical intervention	Change in the papillary area Statistically significantly different in the test group at 3, 6 and 12 months and no statistically significant differences in control group 12 months
43) McGuire and Scheyer [36]	RCT Parallel Perio Health Clinical Research Center in Houston, Texas—University hospital—single setting	21 patients 42 defects 17 female 3 male 51.4 years Non-smokers	1 No Yes	Cultured and expanded autologous fibroblast injections Control group—placebo	Percentage change in papillary height—distance from tip of papilla to base of contact area Significant increase in papillary height in test group at 2 months compared to control but no significant differences at 3–4 months VAS score superior in test group
44) Shapiro [57]	Case report Retrospective University of Montreal Canada Single setting	2 patients 2 females Mean age: 26 years 1 smoker	0 Not mentioned Not mentioned	Repeated gingival curettage every 10 days for 40 days	Clinical appearance and PPD Almost complete regeneration for case 1 at 7 years Increased papillary height but not complete infill for case 2 at 11 months
45) Zanin et al. [71]	Case report University hospital Sao Paulo Brazil Single setting	3 patients 3/9 defects 2 female, 1 male Age range 42–61 years Non-smokers	0 No Yes	Laser—HLT—hemolasertherapy technique	Black triangle height Interdental papilla filled completely at 14 days 4–5 years follow-up showed ‘excellent’ response

Fifteen of the included papers reported on the use of hyaluronic acid including 2 randomised controlled trials [1, 2], 1 cohort study [3], 10 case series [4–10] and 2 case reports [11].

All of the studies reported on the use of an injectable form of HA gel. Eight studies stated the manufacturers of the HA gel with two studies using Hyadent BG, 2 studies using Teosyal, 2 studies using Qi Sheng and one study using Genoss. One study [1] reported on the use of ‘Restylane Lidocaine’ which is a ‘non-animal stabilised cross linked hyaluronic acid filler with a concentration of 20 ml/mg combined with 3% lidocaine’. The remaining studies did not state the specific brand or manufacturer of HA but termed it either as a commercially available HA or simply a hyaluronic acid gel or filler.

The protocols varied for the methods in which the HA was used but broadly in most cases local anaesthetic was applied and the HA gel was injected usually 2–3 mm apically to the deficient papilla. This was repeated usually at 3 weekly intervals. Four studies repeated the intervention at 3 weeks and 6 weeks [1, 2, 5, 9]. In one study, the injections were repeated at 3 weeks and at 3 months [6]. For the two studies by Lee et al. (2006) [8, ], the HA application was repeated every 3 weeks up to five times until the papilla was mostly filled. Çankaya et al. [11] repeated the HA injections every 3 weeks but the end-point of this was not made clear. Pitale et al. [49] reported only 1 application of the HA injection. In the methodology reported by Singh et al. [62], the HA injection was repeated after the first application at the second and third weeks.

The measurements made varied for each study but most commonly included black triangle height, width and surface area, percentage fill or reduction in black triangle area and change in papillary fill. Two studies by [32, 33] also reported the interdental papilla reconstruction rate. All studies reported an improvement in papillary fill at follow-ups ranging from 3 to 6 months with one study [11] reporting outcomes up to 2 years.

There were two randomised controlled trials within the HA group of studies. Abdelerouf et al. [1] carried out an RCT on 10 patients with 36 papilla defects and compared the use of HA filler injection (Restylane lidocaine) with a saline injection in the control group. A series of three injections were given at 3 weekly intervals and the follow-up duration was 6 months. The results showed there was a statistically significant greater mean decrease in black triangle height for the test group at 3 months and a higher patient satisfaction VAS score at 6 months favouring the test (HA) group. However, there was no statistically significant difference at 6 months between groups. Ni et al. [44] carried out a randomised controlled trial on 24 patients with 68 papilla defects which were randomised in a split-mouth design with the test sites receiving a series of 3 HA gel injections at 3 weekly intervals and the control sites receiving saline placebo injections. At the 12-month follow-up, they found that the height of the gingival papilla increased and the area of the black triangle reduced with the HA injections but it was not statistically significantly superior to the use of the saline injection. However, the HA-injected sites grew quicker than the saline group.

Amongst other studies, Abdeloraouf et al. [1] and Spano et al. [64] reported on patient satisfaction using VAS scores and found scores of 45% and 62.5% in improvement of papilla perception respectively.

One study (14) reported an overlay technique involving the creation of sub-periosteal tunnel from the alveolar mucosa to the affected papilla and injecting HA gel into the papilla and into the subperiosteal tunnel as a papillary augmentation technique. The mean papilla fill was 1.75 mm ± 0.5 at 6 months.

2. Platelet-rich fibrin (PRF) (Table 2)

Six of the included papers reported on the use of platelet-rich fibrin. These included 2 randomised controlled trials [12], 1 case series [13] and 3 case reports [14, 15].

When combined with surgical interventions, the two randomised controlled trials [12] within this group of studies demonstrated that PRF provides inferior results compared to the use of a connective tissue graft. However, the use of PRF was associated with less patient morbidity and greater patient satisfaction.

Sharma et al. [59] reported the results of a RCT comparing the use of the Han and Takei surgical papilla reconstruction technique in 20 defects. The control group received the Han and Takei surgical technique with a sub-epithelial connective tissue graft whilst the test group received the Han and Takei technique with PRF inserted into the pouch. At the 3-month follow-up, the mean reduction in CPTP (distance from contact point to alveolar crest) and the mean gain in papillary fill were statistically significant for group 1 compared to the PRF group 2, whilst the PRF group had less morbidity.

Similarly, in a RCT by Singh et al. [62] comparing surgical reconstruction of the interdental papilla in 40 sites with the use of PRF compared to with the use of a connective tissue graft, better results were yielded in the connective tissue graft control group. The increase in interdental papilla height was 3.10 mm (87.3%) and 3.45 mm (95.8%) and the complete papillary fill was 90% and 95% respectively. The patient satisfaction scores were higher in the PRF group.

The 3 case reports [14, 15] and 1 case series [13] showed favourable results with the use of PRF in combination with surgical reconstructive techniques at follow ups ranging from 3 to 6 months.

3. Soft tissue grafting (Table 3)

Fifteen of the included papers reported on the use surgical grafting procedures including 1 randomised controlled trial, 7 case series [20–22, 24] and 8 case reports [16, 25, 27–29, 31, 32]. The surgical procedures utilised included the Beagle’s technique, interproximal tunnelling, coronally advanced flap and the use of subepithelial connective tissue grafts and a free gingival graft.

The Beagle’s surgical technique is described in a case report [10] and involves creating a new papilla with partial thickness incisions palatal to the deficient papilla twice the length of the desired papilla. This is then reflected onto the labial aspect and sutured into position. The study describes a ‘much improved cosmetic situation’ which remained stable for 18 months but with a 4-mm false pocket. There are no numerical outcome measures stated to quantify the results. Chaulker et al. [15] carried out an RCT comparing the effectiveness of the Beagle’s technique to the modified Beagle’s technique in 20 sites with class I or class II papillary recession defects in the maxillary area. The modified Beagle’s technique involves the incisions being carried out on the labial aspect rather than on the palatal side. The results at 6 months found that the modified Beagle’s technique led to increased filling of the papillary defect whilst conversely the Beagle technique led to more shrinkage of the papilla defect. This corresponds to 39.94% reduction in the area of the papillary defect in the Modified Beagle technique group and a 69.55% increase in the Beagle group.

All other studies reported an improvement in papillary fill outcomes at follow-ups ranging from 4 months to 2 years with one paper reporting a 10-year outcome [12].

4. Orthodontic treatment (Table 4)

Four of the included papers reported on the use of orthodontics in the form of 1 cohort study, 1 case series [34] and 2 case reports [35, 36]. Three studies reported an improvement in papillary fill at follow-ups ranging from 1 to 10 years. In a cohort study, Kandasmy et al. [27] analysed casts of participants undergoing orthodontic treatment and compared them to casts of controls. They found that after 18 months, the height of the interdental papillae increased following palatal movement of labially placed or imbricated incisors and following the intrusion of one incisor relative to an adjacent incisor.

In a study of 28 patients presenting with a diastema between the central incisors and associated loss of interdental papilla with one extruded central incisor, the combination of open flap debridement and orthodontic intrusion resulted in improved papilla presence index scores for 23 out of 28 patients at 1 year.

A case report [35] describing a multidisciplinary approach to managing interdental papilla loss between a maxillary right central and lateral incisor involved the use of surgical papilla reconstruction with a connective tissue graft and orthodontic movement. The results demonstrated 3-mm gain in papilla height and correction of the papilla architecture.

A case report [55] described the formation of a non-surgical papilla at the 1 year follow-up after periodontal treatment followed by orthodontic treatment using light force and simultaneous mesial stripping of the incisors.

### Other modalities

Çankaya et al. [11] reported on a RCT comparing surgical reconstruction of the interdental papilla with the use of concentrated growth factor compared to no surgical intervention. The concentrated growth factor was derived from centrifuged blood samples with the protocol described by Qiao et al. [51]. The study reported for the test group a positive correlation with papillary thickness and the filling percentages and between the thrombocyte count and the 6- and 12-month filling percentages. McGuire et al. [36] reported on a randomised controlled trial comparing cultured and expanded autologous fibroblast injections to a placebo and the results found no treatment effect at 4 months. However, the VAS score was superior for the test group.

Other studies with reportedly favourable clinical outcomes describe the use of hemolasertherapy [71] or the use of repeated curettage following acute necrotising ulcerative gingivitis [57].

Due to the high heterogeneity of the studies owing to different study designs, protocols and outcome measures meta-analysis was not possible for any of the studies.

**Fig. 2:**
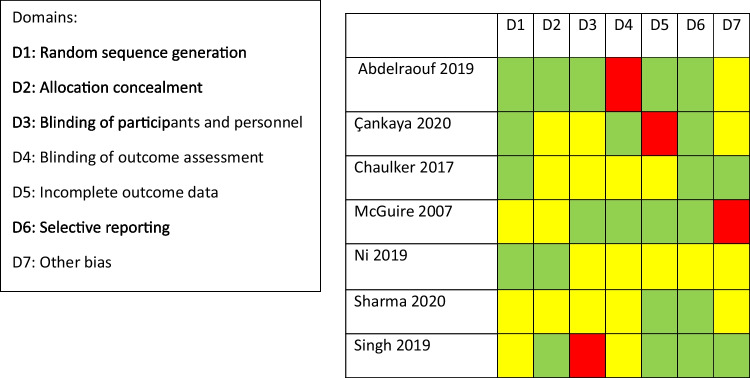
Traffic light system showing risk of bias assessments for RCTs using the RoB2 tool

The Cochrane risk of bias tool was used to assess the risk of bias of the randomised controlled trials. Seven domains were assessed for each of the papers and a traffic light system was used for each category as shown in Fig. 2. Four studies showed a high risk of bias whilst the remainder showed an unclear risk of bias and no papers were deemed to be low risk of bias.

**Fig. 3:**
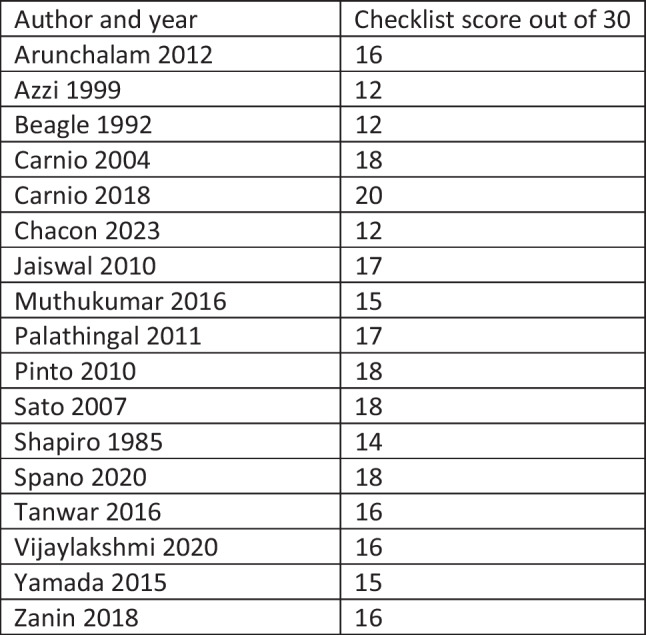
Table showing the CARE checklist score used to assess quality of case reports

The Modified Delphi tool was used to assess risk of bias of case series. All papers included had at least one domain which put the paper into the overall category of high risk of bias. The Newcastle Ottawa scale was used to assess the risk of bias of cohort studies and this ranged from 8 stars [11] to 9 stars [27]. For the case reports, the risk of bias was assessed with the CARE checklist. A score out of 30 was made based on what was included in each case report from the checklist. Figure 3 shows the totals for each paper. Only one paper scored 20 or above. Two papers had a low score of 12 out of 30 and the remainder were in between. This demonstrates that the quality of the case reports did not meet the highest standards based on the checklist.

## Discussion

The main objective of this systematic review was to appraise the literature for the available treatment options for reconstruction of the interdental papilla and to assess how much evidence exists for their efficacy.

Formation of black triangles following non-surgical periodontal therapy and surgical periodontal therapy is an important sequalae of the treatment that clinicians must warn patients about. Loss of interdental papilla in anterior region can also be a frequent cause of dissatisfaction of patients. Cunliffe et al. [19]  reported findings of a patient survey based on perceptions of a series of clinical photographs. The participants ‘ranked black triangles as the third most disliked aesthetic problem after caries and visible crown margins’. Patient’s nowadays have higher aesthetic demands and reconstruction of the interdental papilla is therefore an important aim of periodontal treatment, on which many investigators in different countries have worked for several years but for which no consensus currently exists [54].

Various treatment modalities have been employed for the reconstruction of the interdental papilla. Although most studies included here show improvement of ‘papillary fill’ outcomes at minimum 3 months, it is not possible to make conclusions regarding these techniques due to the lack of long-term data.

The first clear difficulty related to RCTs investigating reconstruction of the interdental papilla is the measurement of the outcome, which ranges from subjective visual assessments to percentage papillary fill and change in black triangle dimensions. Amongst patient-reported outcomes (PROMs), the visual analog scale scores are used to assess patient and clinician perception in the change in the papillary defect. PROMs were only reported in four studies despite them being crucial as these treatments are intended to improve aesthetics and therefore should be used in all studies related to papillary reconstruction.

The second important issue is related to the choice of the ‘control’ group due to the lack of evidence and consensus for a benefit of any treatment and the absence of a gold-standard treatment, limiting interpretation of results.

Use of a connective tissue graft appears to lead to more favourable results compared with PRF when combined with the surgical Han and Takei technique (Singh et al., Sharma et al.), and the modified Beagle technique showed improvements compared with the original Beagle technique [15]. The latter is described in a case report [10] and involves creating a new papilla with partial thickness incisions palatal to the deficient papilla twice the length of the desired papilla. This is then reflected onto the labial aspect and sutured into position. The modified Beagle’s technique involves the incisions being carried out on the labial aspect rather than on the palatal side. Amongst non-surgical interventions, HA injections do not seem to lead to improvements beyond 3 months compared with saline injections [1, 44].

McGuire et al. [36] reported a significant increase in papillary height in the test group with cell transplantation of cultured and expanded autologous fibroblast injections following a papilla priming procedure compared with the placebo control group at 2 months. However, at 3–4 months, there were no significant differences between the two groups. A visual analog scale was used by the participants and examiners and this was superior in the test group receiving the fibroblast injections.

Based on the evidence provided by this systematic review, it seems that the most efficacious intervention for papilla reconstruction is the use of grafting with a sub-epithelial connective tissue graft, whilst non-surgical interventions, including the use of hyaluronic acid, seem to provide less clear benefits.

The surgical techniques involving a connect tissue graft were described in several of the included papers [16, 20, 21, 25, 27, 29, 31–33]. They typically involved semi-lunar incisions, harvesting of a sub-epithelial connective tissue graft from the palate and insertion and coronal advancement of the papilla. Feuillet et al. [20] described a tunnelling technique alongside placement of a connective tissue graft. Carnio et al. [13] described a multidisciplinary case involving a periodontal-orthodontic-restorative approach involving a connective tissue graft. They all reported improvements in the interdental papillary fill. Nemcovsky et al. [42] conducted a case series of 9 patients with 10 defects that underwent surgical papilla augmentation using an advanced papillary flap in combination with a free gingival graft. The results demonstrated an increase in the papilla index score for 8 out of the 10 procedures with a mean increase in PIS of 1.2 ± 0.92 units at 3 months.

These conclusions seem to be in partial agreement with the recommendations proposed by Rasperini et al. [54] in which the treatment on interdental papilla reconstruction was based on the presence of periodontal health or disease. In periodontal health, they advise soft tissue grafts, orthodontics or modification of the restoration. In the presence of periodontal intrabony defects, the surgical management of the defects even with papilla preservation flaps can result in some degree of recession in the interdental area [21]. In the narrative review [54], various techniques are described which are designed to limit recession in the interdental area after periodontal regenerative surgery. This includes the use of enamel matrix derivatives with an envelope coronally advanced flap [72] which is designed to limit supracrestal attachment collapse, increase the space for regeneration and reduce the loss of papilla.

Rasperini et al. [53] described the soft tissue wall technique for regenerative surgery on non-contained intrabony defects in which papilla preservation is used in conjunction with a trapezoidal coronally advanced flap. The authors reported at 12 months an improvement in interdental CAL gain of 7.1 ± 1 mm and a mean recession reduction of 1 ± 0.4 mm. The authors also mention some recent surgical techniques including the connective tissue graft wall, the entire papilla preservation technique [6], use of a connective tissue graft in combination with the single flap approach [67], the modified vestibular incision subperiosteal tunnel access [41] and the non-incised papilla surgical approach [38].

There was a high heterogeneity amongst the studies, mainly due to variations in the protocol, follow-up and outcome measures. Most of the studies included in this systematic review were judged to have either a moderate or high risk of bias. This reduces the quality of evidence and makes it more difficult to make recommendations based on their findings. To limit this, better designed studies need to be conducted. Ideally, these should be randomised controlled trials, with blinding where possible. All studies should be prospective studies rather than retrospective to limit bias also. Outcomes need to be reported more consistently for example with the same papilla indices. Patient-reported outcomes should always be included. Many of the procedures described in the case series and case reports should be further studied and backed up by randomised controlled trials to evaluate their efficacy with limited bias. Longer follow-ups are also needed. The studies need to have a clear inclusion and exclusion criteria especially regarding smoking status. Twelve papers [7, 8, 14, 15, 28, 31, 32, 34, 35] did not report on the smoking status of the participants and this could have affected the outcome.

A strength of the present systematic review is that a comprehensive search strategy was employed using three databases in addition to a manual and cross-reference search. There were no language restrictions and no lower limit of date of publication so all available literature could be systematically assessed. Due to the limited evidence base, we did not restrict this systematic review to RCTs, but tried to be very inclusive in terms of study design and patient numbers. The inclusion of cohort studies, case series and case reports allowed a wider range of studies and data to be incorporated into this systematic review but their lower levels of evidence has resulted in less high-quality data. The reported outcome variables were inconsistent amongst the studies with some papers [28, 29, 36, 39, 40] reporting only a visual assessment of the outcome rather than numerical data, introducing a high level of bias. The evidence strength produced by this review is considerably more robust than what was reported in a systematic review by [22]. They included 8 papers none of which were RCTs, and reported that all of the studies demonstrated ‘positive’ results. They set a limit for publications from 2010 onwards limiting evidence from studies previous to this date whilst this current systematic review did not have a limit to publication date.

## Conclusion

Within the limitations of this systematic review, we can conclude that the loss of the interdental papilla remains an important clinical sequela with significant impact for patients suffering from periodontal disease. Amongst many different treatment modalities available for reconstruction of the interdental papilla, hyaluronic acid injections, PRF, surgical grafting and orthodontics seem to improve outcomes at a minimum 3 months. However, the use of grafting with sub-epithelial connective tissue graft seems to be associated with the most evidence for the longer-term reduction of ‘black triangles’. However, no robust direct comparisons between different techniques are available. Overall, there is insufficient evidence to make recommendations to clinicians and due to the high level of heterogeneity in the studies we cannot draw clear conclusions. Further research in this field should include good-quality RCTs of the most promising treatment modalities with at least a 12-month follow-up, using the appropriate controls and consistent papilla indices and PROMs as outcomes.

### Supplementary Information

Below is the link to the electronic supplementary material.

Supplementary file1 (DOCX 19 KB

## Data Availability

The datasets used during the current study are available from the corresponding author on reasonable request.
