# Supplementary material for: Interdental papilla reconstruction: a systematic review
Source: Clin Oral Investig. 2024 Jan 17;28(1):101. doi: 10.1007/s00784-023-05409-0 (PMC10794407; doi:10.1007/s00784-023-05409-0)
Supplement: Supplementary file 1 — Supplementary file1 (DOCX 19 KB [file 784_2023_5409_MOESM1_ESM.docx]

Supplementary information

Supplementary Table 1: List of excluded papers and reasons for exclusion following full text screening

| **First author and year** | **Reason for exclusion** |
| --- | --- |
| Aubert 1994 | Description of a procedure only |
| Arunachalam 2019 | No results just a description |
| Azzi 1998 | Description of procedure only, follow up not clear |
| Azzi 2001 | Presentation of technique rather than a study, no clear results |
| Brianezzi 2016 | No papilla reconstruction technique- black triangles addressed restoratively |
| Burnice 2020 | No clear results just description of procedure |
| Carranza 2011 | Not long enough follow up |
| Cardaropoli 2005 | Description of technique only |
| Echeverria 1987 | Reflection of a case rather than a study |
| Henriques 2017 | No results |
| Inocencio 2008 | Length of follow up not clear |
| Kim 2014 | Length of follow up not clear |
| Littarru 2021 | Follow up not clear |
| Mandel 2020 | Not long enough follow up |
| Nordland 2008 | Description of procedure only |
| Noverrazs 2020 | No papilla reconstruction technique |
| Prato 1986 | Review |
| Satpathy 2020 | Description of a procedure rather than a study |
| Waki 2006 | No papilla reconstruction technique |
